# Implementation Structure of ERAS Components in Gynecologic Oncology During Early Adoption: A Network-Based Analysis

**DOI:** 10.3390/jcm15134864

**Published:** 2026-06-23

**Authors:** Vasilios Pergialiotis, Dimitrios Haidopoulos, Alexandros Daponte, Dimitrios Tsolakidis, Stamatios Petousis, Ioannis Kalogiannidis, Dimitrios Efthymios Vlachos, Maria Fanaki, Vasilios Lygizos, George Delinasios, Panagiotis Tzitzis, Philipos Ntailianas, Vasilios Theodoulidis, Chrysoula Margioula Siarkou, Nikolaos Thomakos

**Affiliations:** 1 1st Department of Obstetrics and Gynecology, Alexandra Hospital, National and Kapodistrian University of Athens, 11528 Athens, Greece; dimitrioshaidopoulos@gmail.com (D.H.);; 2 Department of Obstetrics and Gynecology, University of Thessaly, 38221 Larissa, Greece; daponte@med.uth.gr (A.D.); gdelis94@gmail.com (G.D.); 3 1st Department of Obstetrics and Gynecology, Papageorgiou Hospital, Aristotle University of Thessaloniki, 54124 Thessaloniki, Greece; 4 2nd Department of Obstetrics and Gynaecology, Ippokratio Hospital, Aristotle University of Thessaloniki, 54124 Thessaloniki, Greece; 5 3rd Department of Obstetrics and Gynaecology, Ippokratio Hospital, Aristotle University of Thessaloniki, 54124 Thessaloniki, Greece; 6 Department of Anesthesia, Alexandra Hospital, 11528 Athens, Greece

**Keywords:** enhanced recovery after surgery, ERAS, gynecologic oncology, perioperative care, network analysis, perioperative pathways

## Abstract

**Objective**: To characterize the structural organization of Enhanced Recovery After Surgery (ERAS) component implementation in gynecologic oncology and determine whether ERAS elements operate as an interconnected perioperative system during early pathway integration. **Methods**: This study represents a secondary analysis of the prospective multicenter Enhanced Recovery in Gynecologic Oncology (ERGO) cohort, including the first 300 consecutive patients undergoing surgery for gynecologic malignancy across five tertiary institutions. Components with prevalence between 5% and 95% were included in a regularized Ising network model to estimate conditional dependencies between pathway elements. Node-level centrality metrics and global network characteristics were calculated to identify structurally influential ERAS components and to describe the overall implementation architecture. **Results**: Thirteen central ERAS components met the predefined prevalence criterion (5–95%) and were included in the conditional dependency network. The estimated network demonstrated substantial inter-component connectivity, indicating that ERAS practices were frequently implemented in coordinated patterns rather than as isolated interventions. Centrality analysis identified postoperative laxatives or chewing gum, tranexamic acid administration, perioperative intravenous fluid management, and avoidance of drain placement as highly connected elements within the network. Early nutritional advancement and postoperative bowel stimulation measures also demonstrated relatively central positions within the recovery-related component cluster. Community detection analysis revealed distinct modules of co-adopted ERAS practices spanning multiple perioperative phases. **Conclusions**: ERAS implementation in gynecologic oncology appears to follow a structured architecture characterized by interconnected perioperative practices rather than independent protocol elements. Understanding these implementation structures may help guide targeted quality-improvement strategies aimed at optimizing ERAS integration in routine clinical practice.

## 1. Introduction

Enhanced Recovery After Surgery (ERAS) represents a multimodal, evidence-based perioperative care pathway designed to reduce surgical stress, accelerate recovery, and standardize perioperative management through coordinated interventions across the preoperative, intraoperative, and postoperative phases [1,2,3]. In gynecologic oncology, ERAS pathways have been increasingly adopted following the publication of ERAS Society guidelines and their subsequent updates, which provide structured recommendations for perioperative care and address challenges related to implementation in routine clinical practice [1,4]. Despite the growing body of evidence supporting improved postoperative outcomes with ERAS programs, real-world implementation remains heterogeneous across institutions [5,6,7,8,9]. Observational studies and international surveys indicate that although many centers report adopting ERAS pathways, consistent adherence to individual pathway components remains variable, reflecting barriers related to institutional infrastructure, multidisciplinary coordination, and local clinical practices [5,9,10].

Successful ERAS adoption typically involves surgeons, anesthesiologists, nursing staff, nutrition specialists, physiotherapists, and hospital administrators working within a coordinated perioperative care structure [11,12,13]. Therefore, ERAS implementation should be conceptualized as a complex adaptive system in which individual components (decision-making procedures) interact with one another, rather than a static checklist of clinical actions. Understanding the structural organization of ERAS implementation is particularly relevant during the early phases of pathway adoption. During these phases, institutions often experience partial implementation in which some ERAS elements are adopted earlier or more consistently than others [14]. Identifying how these components cluster together may help explain patterns of pathway uptake and may provide practical guidance for institutions seeking to improve ERAS integration.

Previous analysis of the ERGO cohort demonstrated heterogeneous adherence across ERAS components and identified patient performance status and surgical complexity as important determinants of protocol compliance [15]. These findings indicated that variability in ERAS implementation is not random but may reflect underlying structured patterns of care delivery across the perioperative pathway [15]. Building on these observations, the present study extends this work by examining the structural relationships between individual ERAS components, aiming to determine whether the pathway functions as an interconnected network of perioperative practices rather than a collection of independent interventions. To accomplish this, we used network-based analytical approaches that allow the evaluation of conditional dependencies between individual variables while accounting for the influence of all other variables in the system, thereby providing a more comprehensive understanding of how ERAS elements interact in routine clinical practice.

## 2. Methods

### 2.1. Study Design, Registration, and Ethical Approval

The present study represents a secondary analysis of the ongoing Enhanced Recovery in Gynecologic Oncology (ERGO) multicenter prospective cohort [15]. The ERGO study was designed to evaluate real-world implementation of ERAS pathways across tertiary gynecologic oncology centers in Greece and is registered at ClinicalTrials.gov (Identifier: NCT06655506) [7,16]. The ERGO study was approved by the Institutional Review Board of Alexandra Hospital (IRB No. 131/2024). In addition, institutional approval was obtained from the local ethics committees or review boards of all participating centers prior to study initiation. All participants provided written informed consent before enrollment and the study was designed in accordance with the Declaration of Helsinki on Ethical Principles for Medical Research Involving Human Participants [17]. ERAS elements were applied as part of standard perioperative care according to contemporary ERAS Society recommendations, and data were recorded consecutively to reflect actual institutional practice rather than controlled trial settings [1]. The current analysis is based on the first 300 enrolled patients within the predefined accrual period.

### 2.2. Eligibility Criteria and Data Collection

The population included in the present analysis has been previously described in our interim ERGO report [15]. Briefly, the ERGO study is a prospective multicenter cohort designed to evaluate real-world implementation of Enhanced Recovery After Surgery (ERAS) pathways in gynecologic oncology practice across tertiary referral institutions in Greece. Adult women scheduled to undergo elective surgical treatment for suspected or confirmed gynecologic malignancy were considered eligible for participation, provided they were deemed clinically suitable to receive perioperative care within an ERAS framework. Eligibility therefore required adequate functional status and the ability to participate in perioperative pathway elements such as early mobilization, nutritional advancement, and postoperative rehabilitation measures. Patients with non-gynecologic metastatic disease or severe functional impairment that would preclude participation in perioperative ERAS interventions were not considered eligible, in accordance with the predefined ERGO study protocol (ClinicalTrials.gov: NCT06655506). Additional exclusions included clinical circumstances in which perioperative management would necessarily deviate from standard ERAS principles due to medical contraindications or urgent surgical indications. Consecutive eligible patients were enrolled prospectively during the predefined study accrual period in order to capture routine clinical practice and minimize selection bias. The multicenter design of the study allowed the evaluation of ERAS implementation across different institutional environments while maintaining standardized definitions of pathway components and perioperative data collection procedures.

Baseline demographic and clinical characteristics were recorded prospectively according to the predefined ERGO study protocol [16]. These included patient age, body mass index, comorbidity profile, American Society of Anesthesiologists (ASA) classification, and Eastern Cooperative Oncology Group (ECOG) performance status. Tumor-related characteristics included primary cancer site, histological subtype, and disease stage based on the International Federation of Gynecology and Obstetrics (FIGO) classification where applicable. Preoperative laboratory parameters reflecting baseline physiological status were also collected, including hemoglobin levels, serum albumin, renal function indices, and inflammatory markers when available.

All ERAS components were defined a priori according to ERAS Society recommendations and were operationalized as binary ERAS-concordant indicators. The components that were used in the present ERAS network represent standardized elements spanning the preoperative, intraoperative, and postoperative phases. Preoperative components focused on patient preparation and optimization and included structured patient counseling regarding perioperative expectations, shortened fasting intervals with clear fluids permitted up to two hours before anesthesia, administration of preoperative carbohydrate loading, avoidance of routine mechanical bowel preparation, and appropriate antibiotic and thromboembolic prophylaxis. Intraoperative components addressed anesthetic and surgical management practices designed to minimize physiologic stress and maintain homeostasis. These included multimodal analgesia strategies aimed at reducing opioid exposure, maintenance of intraoperative normothermia, goal-directed fluid therapy guided by hemodynamic parameters, prophylactic antiemetic administration, and avoidance of routine nasogastric tube placement and prophylactic peritoneal drains unless clinically indicated. Postoperative ERAS elements targeted early functional recovery and included early oral fluid intake, early mobilization within the first postoperative day, early urinary catheter removal, continuation of opioid-sparing multimodal analgesia, early resumption of normal diet, optimization of postoperative fluid balance, and maintenance of adequate glycemic control. Discharge readiness was assessed according to predefined functional criteria including tolerance of oral intake, adequate pain control with oral medication, independent ambulation, and stable vital signs.

For analytical consistency, variables originally reflecting utilization of non-ERAS practices (e.g., drain placement, systemic opioid administration, prolonged intravenous fluids) were inverted so that higher values uniformly represented adherence to ERAS principles. Data were prospectively recorded using standardized electronic case-report forms within the REDCap environment to ensure uniform data capture across participating institutions.

### 2.3. Outcomes

The primary objective of the present analysis was to characterize the conditional dependency structure of ERAS component implementation using network topology metrics derived from a regularized Ising model. In this analytical framework, individual ERAS components are represented as nodes within a network, while the statistical relationships between them are represented as edges. This approach allows for an evaluation of whether ERAS elements are implemented in routine clinical practice as an integrated perioperative system—where several components tend to be delivered together—or whether they function largely as independent decisions driven by individual clinicians. In practical terms, the network model estimates how the presence or absence of a given ERAS component is conditionally associated with other pathway elements after accounting for the entire set of ERAS practices recorded in the dataset.

Within this framework, the analysis focused on identifying ERAS components occupying central positions within the implementation network. These components were identified using node-level centrality indices, including degree, strength, and betweenness. Degree reflects the number of direct connections a component has with other ERAS elements, while strength quantifies the overall magnitude of these connections. Betweenness centrality represents the extent to which a component lies on the shortest pathways connecting other nodes within the network. From a clinical perspective, components with high betweenness centrality occupy bridging positions within the network structure and may connect otherwise less directly related groups of ERAS practices. Accordingly, ERAS practices demonstrating the highest betweenness values were interpreted as structurally important nodes within the implementation network.

Secondary outcomes included additional network-level and node-level characteristics intended to further describe the architecture of ERAS implementation. These included: (i) overall network density and global strength, reflecting the magnitude of inter-component connectivity; (ii) detection of implementation modules using community detection algorithms to identify clusters of ERAS elements that tend to be adopted together; (iii) identification of negatively weighted edges, in which ERAS elements were applied as part of standard perioperative care representing inverse conditional associations between components and; (iv) bootstrap-based assessment of edge and centrality stability to evaluate the robustness of the estimated network structure.

### 2.4. Statistical Analysis

All analyses were conducted in R (Posit team (2025). Rstudio (R-4.6): Integrated Development Environment for R), using an interim cohort of the first 300 consecutively enrolled patients. The analyses were prespecified as exploratory and focused on describing the implementation structure rather than estimating causal effects. ERAS components were screened for sufficient variability; variables with prevalence outside the 5–95% range were excluded from network estimation because near-constant indicators do not contribute stable conditional dependence structure. The ERAS implementation structure was modeled as a regularized Ising network (binary Markov random field), estimated via L1-penalized neighborhood selection with Extended Bayesian Information Criterion model selection. The dplyr package was used for data cleaning and the IsingFit package for Ising modeling. Network adjacency and edge weights were visualized using the qgraph package, with edges representing conditional associations between ERAS components after adjustment for all others. For visualization purposes, a prespecified threshold (|β| ≥ 0.10) was applied to the figure to improve interpretability; all network metrics were calculated using the full estimated network. Node-level metrics were computed using standard centrality indices including degree, strength (sum of absolute edge weights), and betweenness (frequency a node lies on shortest paths between other nodes). Centrality estimates and comparisons were generated using functions available from the qgraph and igraph libraries. The same libraries were used to compute global network metrics, including edge count, density, mean absolute edge weight, global strength, and clustering coefficient. Implementation modules (communities) were detected using modularity-based community detection. Edges with negative conditional weights were identified and summarized descriptively as inverse conditional associations between ERAS components. Bootstrap procedures were used to evaluate the network robustness through assessment of edge-selection stability and stability of node centrality estimates across repeated resampling iterations.

## 3. Results

Briefly, the present analysis included the first 300 consecutively enrolled patients. Of the ERAS elements recorded in REDCap, 13 components met the predefined inclusion criteria of 5–95% prevalence and were therefore eligible for network estimation. The eligible components included preoperative, intraoperative, and postoperative domains, allowing evaluation of ERAS implementation as an integrated perioperative system rather than isolated checklist items that span specific timepoints of the perioperative period.

### Overall Network Architecture

**Figure 1:**
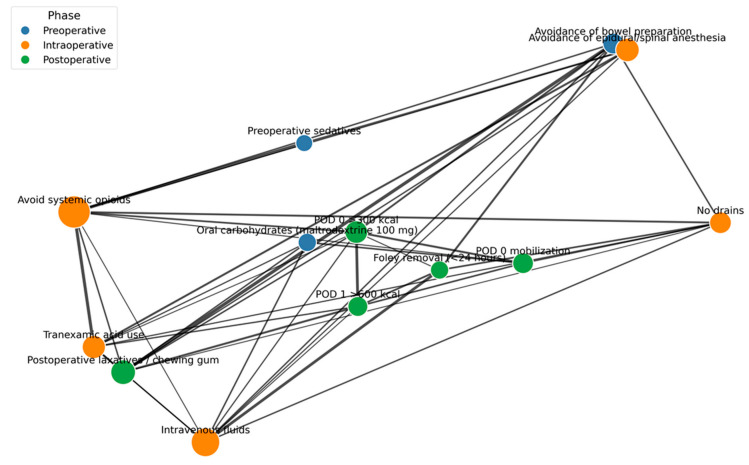
Conditional dependency network of ERAS component implementation. Nodes represent ERAS components meeting the 5–95% prevalence criterion. Edges represent conditional associations estimated using a regularized Ising model. Node colors indicate perioperative phase.

The produced conditional dependency network consisted of 13 nodes connected by 43 edges, thus exceeding the predefined display threshold (|β| ≥ 0.10). This observation suggests that ERAS implementation in this series of patients consisted of densely interconnected components (Figure 1). This was also supported by the network density and global strength metrics analyses that supported substantial inter-component connectivity (Table 1).

**Figure 2:**
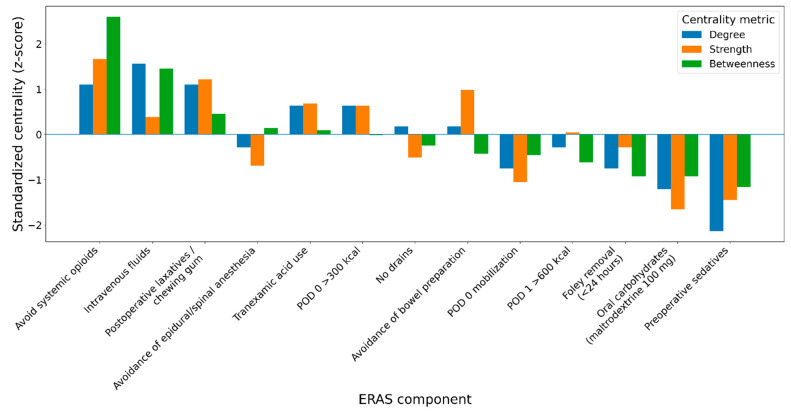
Centrality profile of ERAS components. Standardized (z-score) centrality indices for ERAS components included in the conditional dependency network. Values represent standardized centrality estimates relative to the mean centrality of all nodes within the network. Positive values indicate above-average centrality, whereas negative values indicate below-average centrality.

Node-level centrality analysis identified avoidance of systemic opioids, perioperative intravenous fluid management, and postoperative laxatives or chewing gum as the most central components within the implementation network (Table 2). These elements demonstrated consistently high values across multiple centrality metrics, indicating extensive connectivity and prominent positions within the overall network structure. Tranexamic acid administration, avoidance of bowel preparation, and achievement of early nutritional targets (POD 0 > 300 kcal) also demonstrated relatively high centrality profiles. In contrast, preoperative sedatives and oral carbohydrate loading occupied more peripheral positions within the network (Table 2). The standardized centrality profile (Figure 2) illustrates this differential structural positioning across components.

Global network metrics further characterized the overall implementation architecture of the network (Table 1). The estimated network contained 43 conditional associations among the 13 included ERAS components, corresponding to a density of 0.551. This finding suggests that implementation of individual ERAS elements was highly interconnected, with many practices tending to be adopted alongside others rather than in isolation. The mean absolute edge weight was 0.368, and the overall global strength of the network was 15.817, indicating substantial connectivity across the pathway. The weighted clustering coefficient was 0.219, indicating a moderate tendency for ERAS components to form locally interconnected groups of practices. Community detection analysis identified four distinct modules with a modularity coefficient of 0.199, suggesting the presence of recognizable implementation clusters while maintaining substantial connectivity across the overall network. Considering these findings, we proceeded to community detection analysis to further characterize the internal organization of the ERAS implementation network. This analysis identified distinct modules of co-adopted ERAS elements, indicating that several pathway components tended to cluster together within the overall network structure (Supplementary Figure S1). Community detection analysis identified four distinct implementation modules within the network (Supplementary Figure S1). Collectively, these modules encompassed ERAS practices spanning preoperative preparation, intraoperative management, and postoperative recovery domains. Although the detected communities remained interconnected within the overall network, the observed modular structure suggests that certain ERAS elements tended to cluster together as part of the recognizable implementation patterns.

Analysis of edge directionality demonstrated that the majority of the conditional associations between ERAS components were positive (Supplementary Figure S2). Positive edges indicate that the presence of one ERAS element was conditionally associated with a higher probability of implementation of another element after accounting for the remaining components included in the network model. These positive associations constituted the predominant type of connection within the network, reflecting the overall pattern of interrelationships among ERAS practices in the present cohort. This predominance of positive associations suggests that ERAS practices were generally adopted in a complementary manner rather than independently. In contrast, only a small number of negatively weighted edges were identified (Supplementary Table S3), and these associations were generally weaker than the strongest positive connections. Negative edges indicate inverse conditional relationships between pairs of components after adjustment for the remainder of the network. Their relative scarcity suggests that potentially competing implementation patterns were uncommon within the present cohort. Bootstrap resampling procedures were performed to evaluate the robustness of the estimated network structure and the stability of the identified central components. These analyses assess the extent to which the observed network characteristics remain consistent when the dataset is repeatedly resampled, thereby providing an indication of the reliability of the estimated associations. Strength centrality demonstrated the highest stability across bootstrap iterations, with a median Spearman correlation of 0.92 (95% bootstrap interval 0.81–0.97), indicating that the relative importance of individual ERAS components remained highly consistent across resampled datasets. Degree centrality also demonstrated good stability (median correlation 0.82, 95% bootstrap interval 0.54–0.95), whereas betweenness centrality showed lower stability (median correlation 0.55, 95% bootstrap interval 0.08–0.85). In addition, the strongest conditional associations between ERAS components were consistently retained across bootstrap iterations, supporting the reproducibility of the principal network connections. Taken together, these findings suggest that the overall topology of the network and the identification of the most central ERAS components were robust to sampling variability within the present dataset (Supplementary Figure S3; Supplementary Table S2).

## 4. Discussion

### 4.1. Principal Findings

In the present network analysis of ERAS component implementation, we identified a structured pattern of conditional dependencies among perioperative care elements. Rather than appearing as independent clinical actions implemented in isolation, ERAS components demonstrated substantial interconnectivity within the estimated network structure. This pattern indicates that, in routine clinical practice, several ERAS practices tend to be adopted together, forming coordinated clusters of perioperative management decisions. Such clustering supports the concept that ERAS pathways function as integrated perioperative care systems in which multiple elements are implemented in conjunction with one another, rather than as simple checklists of independent interventions. The relatively high network density observed in the present study suggests that ERAS components remained extensively interconnected even after adjustment for all other variables included in the model, supporting the concept of ERAS as an integrated perioperative care pathway rather than a collection of isolated practices.

Node-level centrality analysis revealed that only a limited number of ERAS components occupied highly central positions within the implementation network. Postoperative laxatives or chewing gum, avoidance of systemic opioids, and perioperative intravenous fluid management demonstrated the highest overall centrality profiles across multiple metrics, indicating that these practices occupied particularly well-connected positions within the implementation network. Tranexamic acid administration and avoidance of bowel preparation also demonstrated substantial connectivity with other ERAS components. These elements were conditionally associated with multiple other ERAS practices and occupied positions linking otherwise distinct parts of the network. Their centrality suggests that they are closely embedded within broader patterns of ERAS implementation and may represent key markers of more comprehensive pathway adoption. From a clinical perspective, these findings indicate that certain perioperative practices are particularly interconnected with other ERAS elements and may therefore play an important role within the overall implementation structure.

The central position of several intraoperative practices, including intravenous fluid management, tranexamic acid administration, and analgesic strategies, highlights the important contribution of anesthesiology to ERAS implementation. These components demonstrated strong conditional associations with multiple ERAS elements and occupied structurally central positions within the network, suggesting that their implementation frequently occurs alongside other perioperative practices. Given that these interventions are largely coordinated by anesthesiology teams during the intraoperative period, their central network positions suggest that they are closely integrated with other ERAS practices across the perioperative pathway.

Community detection analysis further demonstrated that ERAS components organized into distinct modules of co-adopted practices spanning the preoperative, intraoperative, and postoperative phases of care. In practical terms, this means that certain ERAS elements tended to be implemented together as part of coherent groups of clinical practices rather than being adopted independently. For example, several postoperative recovery measures, such as early mobilization, early nutritional advancement, and bowel stimulation strategies, clustered within the same module, suggesting that these elements are frequently delivered together as part of coordinated postoperative management. Similarly, intraoperative practices related to anesthetic management and fluid therapy tended to cluster within their own module, highlighting their close structural relationships within the network. Notably, the identified modules broadly corresponded to the chronological phases of perioperative care, suggesting that ERAS implementation may be organized into interconnected domains that reflect different stages of the perioperative care pathway.

Importantly, bootstrap resampling analyses confirmed the stability of the centrality estimates and the strongest conditional associations within the network. Strength centrality demonstrated high stability across resampled datasets, while degree centrality showed good stability and betweenness centrality exhibited lower but acceptable stability. In addition, the strongest network connections were consistently retained across bootstrap iterations. Together, these findings indicate that the overall structure of the network, and particularly the identification of the most central ERAS components, were robust to sampling variability within the present dataset.

### 4.2. Comparison with Existing Literature

In our previous study we evaluated adherence to ERAS and observed that it was strongly associated with patient performance status and surgical complexity, suggesting that implementation may be influenced by structured practice patterns [15]. The present study builds on these observations by examining the structure of implementation at the level of individual ERAS components rather than patient-level patterns. Together, these analyses suggest that during early integration of ERAS pathways, components may organize both into reproducible patient-level implementation patterns and into a structured network of interrelated perioperative practices.

Our findings align with prior work showing that ERAS uptake in gynecologic oncology is heterogeneous across institutions, but add a structural layer by demonstrating that variability is not simply based on an absolute threshold that determines optimal rates of compliance but rather follows reproducible configurations of component delivery. Large survey-based studies have consistently reported incomplete and uneven implementation at the center level. Specifically, in a German NOGGO-AGO survey, authors observed that fewer than half of hospitals reported adherence to >70% of ERAS elements for primary cytoreductive surgery, and only a small minority reported very high adherence thresholds [18]. Similarly, a Europe-wide ESGO/ENYGO lead survey found that while many centers reported ERAS implementation, adoption and practice patterns varied substantially across participating institutions, underscoring the gap between guideline dissemination and routine delivery [19]. Another study that included 153 healthcare providers found that only 20.3% reported practicing ERAS in major surgery [20]. The main barriers were lack of resources and coordination, limited staffing and funding, and insufficient knowledge or awareness of ERAS. The authors concluded that wider ERAS adoption requires targeted education, stronger institutional support, better multidisciplinary collaboration, and organizational strategies to address logistical and workforce constraints. Several other studies have been published in various surgical specialties that also underline the importance of strong leadership, active multidisciplinary collaboration, structured education, and the use of standardized protocols supported by continuous audit and feedback [9,21,22,23]. However, common barriers arise from limited awareness or understanding of ERAS principles, resistance to changing established perioperative practices, insufficient institutional support, and resource constraints, including staffing pressures and lack of protected time. Variability in clinician receptivity and competing organizational priorities further confer to the inconsistent uptake variable. Nevertheless, it should be noted that the methodology of all these studies is simply based on aggregated adoption rates; thus, it cannot directly describe how specific components cluster together in real-world care.

Single-center and network-wide quality-improvement initiatives have demonstrated that structured ERAS programs can be implemented and can change practice, supporting the clinical plausibility of bundled pathway delivery [23,24,25,26,27]. However, most prior reports evaluate implementation through composite compliance scores or pre/post comparisons of selected elements, which may obscure the internal “architecture” of ERAS delivery. Multiple barriers to ERAS adoption have been described, including resistance to change among clinicians, limited institutional resources, insufficient staffing, and challenges in multidisciplinary coordination across perioperative teams. These factors can significantly influence the ability of institutions to implement ERAS pathways consistently in routine clinical practice. Prior implementation studies and reviews have highlighted that successful ERAS adoption requires not only evidence-based guidelines but also structured change-management processes, institutional leadership, and continuous audit systems to support practice transformation [23,26,28]. In gynecologic oncology specifically, recent analyses have further emphasized that differences in institutional infrastructure, organizational culture, and clinical workflows may contribute to heterogeneous ERAS implementation and variations in pathway adherence across patient populations [6,29]. Although these organizational and behavioral determinants are likely to influence ERAS adoption, their detailed evaluation was outside the scope of the present study. However, the current analysis serves as a structural exploration that may help generate hypotheses on how such barriers interact with ERAS implementation patterns in routine clinical practice.

### 4.3. Strengths and Limitations

The present study is based on a prospectively collected multicenter cohort including consecutive patients undergoing surgery for gynecologic malignancies, which enhances the pragmatic nature of the dataset and reflects real-world implementation of ERAS pathways across different institutions. All components were defined using standardized ERAS-concordant indicators based on international guideline recommendations, thereby ensuring the consistency and reproducibility of the analysis. By introducing component-level network analysis with previously described ERAS adherence indicators [15], the study provides complementary perspectives that help characterize how ERAS pathways are operationalized in routine clinical practice. This integrated approach allows identification of both recurring patterns of ERAS delivery and the structural relationships between individual pathway components.

It should be noted, however, that some limitations should be acknowledged. Firstly, external validation is required. Although the study included patients from five tertiary centers, all institutions were located within a single national healthcare system, and the identified implementation structures should therefore be confirmed in independent cohorts and different healthcare environments. Second, institution-level implementation patterns may have influenced the observed network structure. Formal center-specific network analyses were not performed because the available sample size within individual institutions was insufficient for stable network estimation. Because patients were recruited from five institutions, some observed component relationships may partly reflect shared institutional practice patterns. However, the available sample size within individual centers was insufficient to permit stable center-specific network estimation. Moreover, correlation with organizational or behavioral barriers to ERAS adoption, such as institutional infrastructure, multidisciplinary coordination, or clinician-level factors, was not performed, as this was outside the scope of the ERGO study. Although these determinants likely influence pathway implementation, their evaluation was beyond the objectives of this analysis. Finally, the available sample size limited the ability to perform additional sensitivity analyses that could further explore the robustness of the observed implementation structure. Larger datasets would allow the application of multilevel modeling approaches to investigate the impact of surgical complexity, tumor type, and institutional characteristics on the network structure, thereby enabling a more detailed evaluation of how the ERAS implementation topology varies across these clinical domains.

## 5. Conclusions

Enhanced Recovery After Surgery (ERAS) implementation in gynecologic oncology appears to follow a structured perioperative architecture rather than occurring as a collection of independent clinical actions. The findings of the present study indicate that multiple ERAS practices tend to be implemented in interconnected patterns across the perioperative pathway, suggesting that ERAS functions in routine clinical care as an integrated system of perioperative interventions. Within this structure, practices related to intraoperative management, postoperative bowel recovery, nutritional advancement, and avoidance of drain placement occupied central positions within the implementation network. These findings highlight the importance of coordinated perioperative care and suggest that successful ERAS adoption depends not only on implementation of individual recommendations but also on integration of groups of related practices across different phases of care. More broadly, the results provide insight into how ERAS pathways are actually implemented in real-world settings and may help identify practical targets for implementation efforts aimed at improving pathway integration. Integrating structured perioperative audits and expanding feedback and educational initiatives for clinical teams may further strengthen the coherence of ERAS implementation and help consolidate a more robust and consistently applied perioperative care pathway.

## Figures and Tables

**Table 1:** Global network metrics of ERAS implementation. Summary of global network characteristics describing the overall structure of ERAS component connectivity. Network density represents the proportion of possible connections present between ERAS components. Global strength reflects the cumulative magnitude of conditional associations across the network. The clustering coefficient indicates the tendency of components to form locally interconnected clusters, while modularity quantifies the degree to which the network organizes into distinct implementation modules.

Metric	Value
Number of patients	300
Components included	13
Number of edges (|β| ≥ 0.10)	43
Network density	0.551
Mean absolute edge weight	0.368
Global strength (sum of absolute edge weights)	15.81
Weighted clustering coefficient	0.219
Modularity	0.199
Detected communities	4

**Table 2:** Node-level metrics of ERAS components. Node-level centrality measures for ERAS elements included in the conditional dependency network. Strength represents the sum of absolute edge weights connected to the node. Betweenness reflects the proportion of shortest paths between other nodes that pass through the component, indicating its potential role as a bridging element within the network. Closeness represents the inverse average distance to all other nodes in the network.

ERAS Component	Prevalence (%)	Degree	Strength	Betweenness	Closeness
Postoperative laxatives/chewing gum	91.1	9	3.334	0.062	0.8
No drains	36.1	7	2.058	0.035	0.706
Tranexamic acid use	39.7	8	2.94	0.048	0.75
Intravenous fluids	87.8	10	2.72	0.1	0.857
Avoidance of epidural/spinal anesthesia	74.1	6	1.924	0.05	0.667
POD 0 > 300 kcal	71.9	8	2.902	0.044	0.75
Avoidance of long sedatives	42.9	2	1.368	0	0.48
Avoid systemic opioids	56.3	9	3.665	0.144	0.8
Avoidance of bowel preparation	79	7	3.159	0.028	0.706
POD 1 > 600 kcal	85.6	6	2.472	0.021	0.632
POD 0 mobilization	71.9	5	1.658	0.027	0.632
Oral carbohydrates (maltrodextrine 100 mg)	75.3	4	1.211	0.009	0.571
Foley removal (<24 h)	50	5	2.223	0.009	0.6

## Data Availability

The data that support the findings of this study are not publicly available due to restrictions related to privacy and ethical considerations but are available from the corresponding author upon reasonable request.

## Supplementary Materials

The following supporting information can be downloaded at: https://www.mdpi.com/article/10.3390/jcm15134864/s1, Table S1: Bootstrap edge selection stability in the ERAS implementation network; Table S2: Bootstrap stability of centrality metrics; Table S3: Center-level descriptive network characteristics; Figure S1: Community structure of the ERAS implementation network; Figure S2: Positive and negative conditional associations between ERAS components; Figure S3: Bootstrap stability of centrality estimates.
