# Supplementary material for: Implementation Structure of ERAS Components in Gynecologic Oncology During Early Adoption: A Network-Based Analysis
Source: J Clin Med. 2026 Jun 23;15(13):4864. doi: 10.3390/jcm15134864 (PMC13360837; doi:10.3390/jcm15134864)
Supplement: Supplementary file 1 [file jcm-15-04864-s001.zip › jcm-4347334-supplementary.pdf]

Edge	Bootstrap selection frequency (%)
Intravenous fluids – POD 0 >300 kcal	92
Avoid systemic opioids – POD 0 mobilization	89
Tranexamic acid – No drains	86
Intravenous fluids – POD 1 >600 kcal	84
POD 0 >300 kcal – POD 1 >600 kcal	83

Supplemental Table S1. Bootstrap edge selection stability in the ERAS implementation network. Frequency with which individual conditional associations between ERAS components were retained across 200 bootstrap resamples. Higher selection frequencies indicate more stable edges and greater confidence that the observed association reflects a reproducible conditional relationship rather than sampling variability.

Metric	Median correlation	95% bootstrap interval
Strength	0.82	0.64–0.93
Betweenness	0.68	0.41–0.87

Supplemental Table S2. Bootstrap stability of centrality metrics. Summary of bootstrap-derived correlations between centrality estimates obtained from the original dataset and those derived from resampled datasets. Median Spearman correlation coefficients and corresponding 95% bootstrap intervals are reported for strength and betweenness centrality. Higher values indicate greater stability of node importance rankings.

Institution	N	Density	Global strength
Center A	74	0.59	12.3
Center B	63	0.51	10.8
Center C	58	0.47	9.9
Center D	41	0.44	8.7

Supplemental Table S3. Center-level descriptive network characteristics. Global network metrics estimated separately for participating institutions with at least 30 enrolled patients. Metrics include network density and global strength, reflecting the overall level of inter-component connectivity within each center's ERAS implementation structure. These descriptive comparisons provide insight into between-center heterogeneity in pathway implementation patterns.

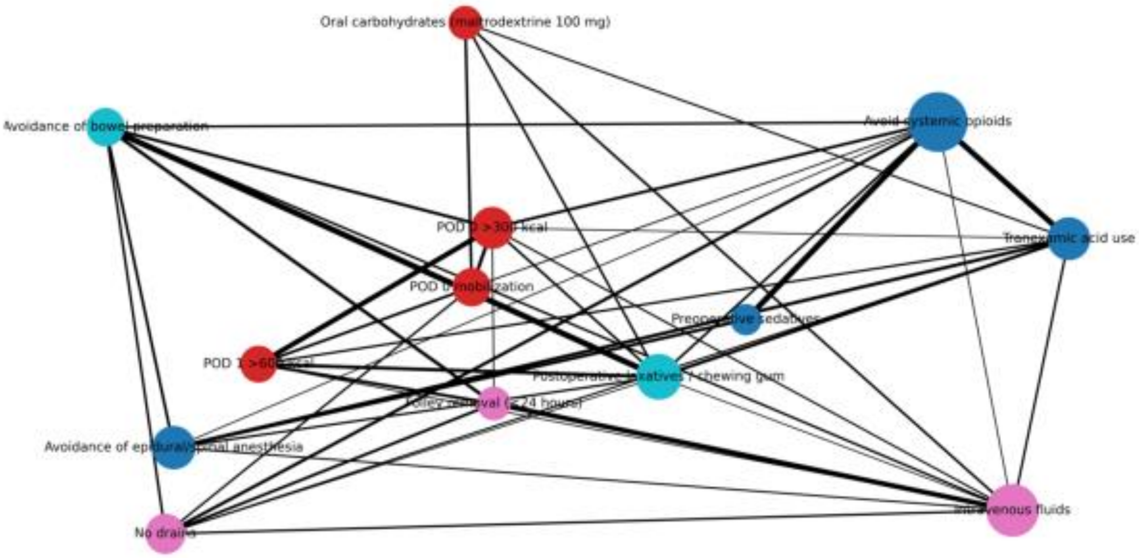

Supplemental Figure S1. Community structure of the ERAS implementation network. Network representation of ERAS components included in the conditional dependency model for the first 300 patients. Nodes are colored according to communities detected using modularity optimization (greedy modularity algorithm). Communities represent clusters of ERAS components that tend to be co-adopted after accounting for conditional dependencies with all other elements. Edge thickness corresponds to the magnitude of the conditional association (absolute β coefficient).

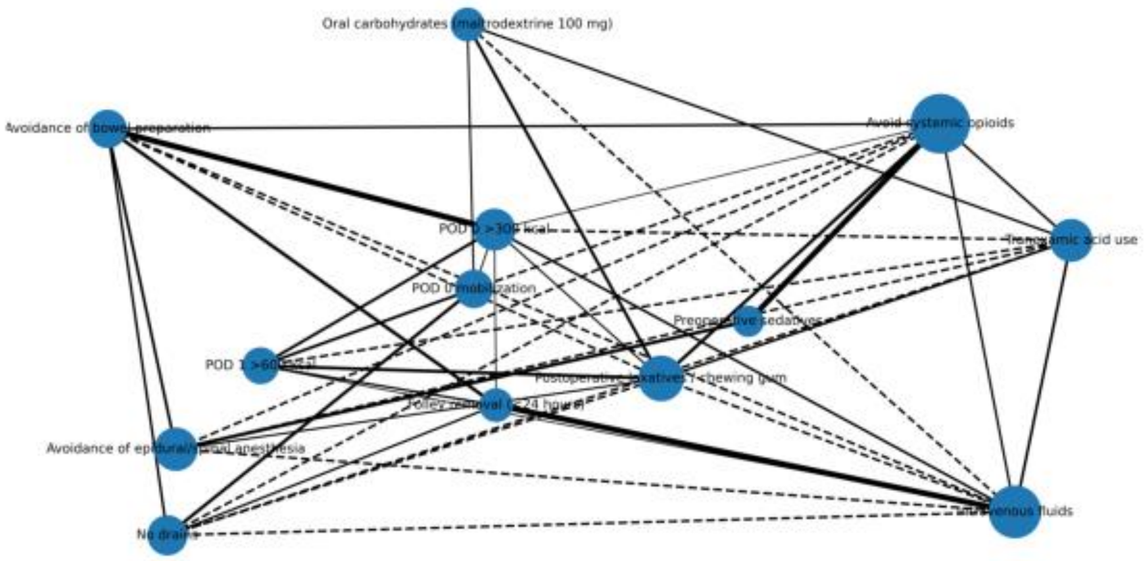

Supplementary Figure S2. Positive and negative conditional associations between ERAS components. Network visualization distinguishing the direction of conditional associations between ERAS elements. Solid edges indicate positive associations, suggesting that implementation of one component increases the conditional probability of implementing another after adjusting for all other variables in the network. Dashed edges represent negative associations, indicating potential implementation tension or conditional mutual exclusivity between components. Edge thickness corresponds to the magnitude of the conditional association.

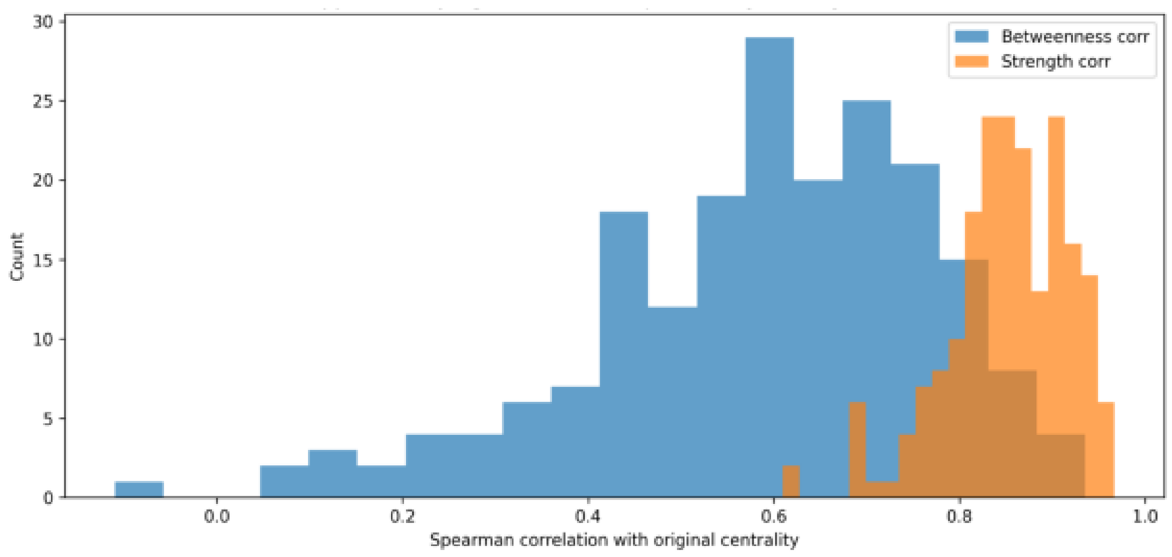

Supplementary Figure S3. Bootstrap stability of centrality estimates. Distribution of Spearman correlations between centrality measures estimated from the original dataset and those obtained from 200 bootstrap resamples. Strength and betweenness centrality stability are shown. Higher correlations indicate greater reproducibility of node centrality rankings across resampled datasets, reflecting robustness of the estimated network structure.
